# Increased Anxiety, Akathisia, and Suicidal Thoughts in Patients with Mood Disorder on Aripiprazole and Lamotrigine

**DOI:** 10.1155/2015/419746

**Published:** 2015-10-05

**Authors:** Milena Pereira Pondé, Antonio Carlos Cruz Freire

**Affiliations:** BAHIANA School of Medicine and Public Health, Avenida Dom João VI, No. 275, Brotas, 40290-000 Salvador, BA, Brazil

## Abstract

*Introduction*. Akathisia affects around 18% of patients with bipolar disorder treated with aripiprazole and may worsen when aripiprazole is combined with lamotrigine and antidepressants. *Case*. This paper reports on two clinical cases involving patients with a diagnosis of mood disorder who developed severe akathisia, anxiety, and suicidal ideation while using a combination of aripiprazole, antidepressants, and lamotrigine. *Discussion*. We recommend that patients with a mood disorder taking multiple drugs should begin aripiprazole therapy with low doses and be monitored for the development of akathisia, increased anxiety, or suicidal thoughts. The appearance of these limiting side effects requires discontinuation of the drug.

## 1. Introduction

Aripiprazole is the first antipsychotic drug of a group of new drugs with a peculiar pharmacodynamic profile. It is a dopamine D2 receptor partial agonist, as well as a 5-HT1A receptor partial agonist and a 5-HT2A receptor antagonist [1]. The partial agonistic effect of aripiprazole on 5-HT1A receptors may be associated with improvements in anxiety, depression, and negative symptoms and a reduction in extrapyramidal symptoms [2]. Aripiprazole is widely used to treat patients with bipolar disorder and is indicated for the treatment of depressive symptoms and as a mood stabilizer, with the advantage of having no negative effect on patients' metabolic profile. Aripiprazole is approved in the US and in Europe for the treatment of the acute manic and mixed episodes associated with bipolar I disorder. Clinical trials have shown aripiprazole to be clinically effective in terms of response and remission rates and in preventing relapses both when used for managing acute mania and as a long-term maintenance treatment. The lack of any sedative effect does not affect the efficacy of aripiprazole in controlling mania and restlessness [3]. Aripiprazole monotherapy resulted in some improvements in core depressive symptoms in patients with bipolar I disorder who had more severe depression; however, the difference in relation to the placebo group was not statistically significant [4]. Adjunctive aripiprazole treatment in patients with depression and a history of an inadequate response to antidepressants was associated with a reduced suicidality rate in a group of subjects at no significant risk [5]. The association of aripiprazole for depressive patients with anxiety or atypical characteristics resulted in a statistically significant improvement in depressive symptoms compared to placebo, the adverse effects being comparable in the patients with anxious and nonanxious depression and with typical and atypical depression. The rates of akathisia and weight gain in patients on aripiprazole treatment did not differ between subgroups, leading Trivedi et al. to conclude that adjunctive aripiprazole is an effective treatment for patients with major depression presenting with either anxiety or atypical features [6].

Akathisia is a known side effect of aripiprazole that worsens when the drug is associated with lamotrigine or antidepressants. Akathisia is the most common of all the extrapyramidal side effects in the population using antipsychotics [7]. It consists of a subjective feeling of restlessness or nervousness, a need to move about, and an inability to relax that may be accompanied by signs of restlessness such as leg swinging while sitting, rocking from foot to foot, or swaying back and forth [7]. Symptoms generally appear 5–10 days after initiating use of a traditional antipsychotic drug or after an increase in its dose [7]. The prevalence of akathisia was 18% in patients with bipolar I disorder using aripiprazole and led to discontinuation of the drug in 2.3% of patients, which was greater than the discontinuation rate for akathisia in a group of patients with schizophrenia or schizoaffective disorder (1.2%) [8]. A study conducted to test the combination of lamotrigine and aripiprazole for patients with bipolar I disorder reported akathisia to be the most common side effect, with a prevalence of 10.8% in the group using aripiprazole and lamotrigine compared to 6.1% in the lamotrigine and placebo group [9]. A good response has been found with the association of low doses of aripiprazole (a mean of 4.2 mg/day) with antidepressants for treatment-resistant depression, with a prevalence of akathisia of around 22% [10].

Recent case reports have suggested that akathisia constitutes a limiting factor to the use of aripiprazole. Saddichha et al. reported a case in which the use of aripiprazole was associated with acute dystonia, akathisia, and parkinsonism in a single patient [11]. Primeau et al. reported serotonin toxicity in aripiprazole augmentation, highlighting the risk of combining aripiprazole with antidepressants [12]. One case report described the development of aripiprazole-induced agitation following discontinuation of clozapine [13], while another reported aripiprazole-associated bruxism, akathisia, and parkinsonism in a bipolar patient [14]. Chem and Liou reported acute aripiprazole-associated dystonia, akathisia, and parkinsonism in a patient with bipolar I disorder [10].

## 2. Case

JSM, a 29-year-old female business manager, was extremely reserved and shy as a child living in a difficult family environment with a verbally abusive father. At 19 years of age she began analysis to cope with the stress of studying for her university entrance exams. At 21, she was prescribed fluvoxamine because of prominent obsessive symptoms. These symptoms disappeared, and she felt well, cheerful, and more energetic than usual for around 10 days. However, two months later she attempted suicide by taking benzodiazepines because of extreme anguish. Her family reported that at that time the patient had been very irritable, verbally abusive, and complaining of anguish that led her to suicidal thoughts and some suicide attempts. At 23, her progress at university was mediocre since she was unable to concentrate and would spend an entire week lying in bed, haunted with suicidal thoughts. In addition, since adolescence she has had dissociative seizures during which she cries a lot, shouts, contorts her hands and feet, and is unable to walk. At 26, after the end of a relationship, she became extremely apathetic and prostrate and had suicidal thoughts for about two months. She initiated use of venlafaxine but there was no significant improvement. The dissociative seizures intensified. Suspecting epilepsy, a neurologist prescribed lamotrigine. Nevertheless, an electroencephalogram (EEG) and video EEG monitoring failed to confirm the diagnosis of epilepsy. Symptoms of psychosis developed and her depression deepened. The antidepressant was switched to duloxetine (60 mg), which was then combined with mirtazapine (30 mg), and the dose of lamotrigine was increased to 400 mg/day. The psychotic symptoms worsened and she began to have suicidal thoughts and to self-mutilate. Aripiprazole (5 mg) was added but there was no improvement; instead the symptoms of psychosis, anxiety, insomnia, and anguish worsened and akathisia developed. After about 20 days, the dose of the drug was adjusted to 10 mg; however, the aforementioned symptoms worsened and the psychotic experiences intensified. The dose was then increased to 15 mg and the patient was now no longer able to keep still for more than five minutes at a time. She continued to self-mutilate, the suicidal thoughts intensified, and she attempted defenestration. The symptoms began to improve gradually when all the medication was stopped and clozapine was introduced. Clozapine was gradually increased up to a maximum dose of 125 mg/day and resulted in remission of the acute symptoms after 15 days. The patient has remained stable for around 20 months, with relapses of mild-to-moderate depressive symptoms for periods of 10 days or less. The diagnostic hypothesis in the case of this patient is recurrent depression or type II bipolar disorder in view of the suspected episode of hypomania at the age of 21 years.

OLP, a 56-year-old male fashion designer, had a very unhappy childhood, with few friends and many arguments at home. At university, he underwent a substantial change, becoming extroverted, making a lot of friends, and travelling extensively. As a professional, he was very creative, had many friends, and enjoyed going out. His father, an alcoholic who was extremely verbally abusive, committed suicide at 60 years of age. A nephew was diagnosed with depression. About 13 years ago, OLP began to isolate himself from his friends and to become very nervous whenever he was with a lot of people. He had difficulty concentrating, lost his enthusiasm for work and his creativity, and seemed to have lost his love for life. During that same period, he felt irritated and was bothered by noise, often getting into arguments with his neighbors for that reason. At work, he would also argue with colleagues, becoming angry easily and later regretting his outbursts. Since that time he has been undergoing psychotherapy and for the past eight years has undergone psychiatric treatment. He has used venlafaxine, mirtazapine, these two drugs together, and bupropion; however, there has been no remission of the depressive symptoms. After two months on bupropion (300 mg/day) and lamotrigine (100 mg/day) without any satisfactory improvement, aripiprazole (10 mg/day) was added. After five days of this combination, he developed major psychomotor agitation, accompanied by increased anxiety and restlessness, and akathisia that did not allow him to sit still for even three minutes at a time. He was sleepless at night even when taking 120 mg of flurazepam and complained of marked tiredness and muscle pain. In addition, his suicidal thoughts intensified and he experienced extreme anguish. He was given 50 mg of clozapine and all the other drugs were discontinued. After 10 days there was a significant improvement in his sleeping, allowing him to return to work. In addition, his psychomotor agitation decreased and there was a partial improvement in his feelings of anguish. Nevertheless, depressive symptoms remained: negative thoughts, extreme apathy, indisposition, and poor concentration. He was prescribed imipramine 150 mg and lithium 450 mg. He remained stable, with mild depressive symptoms after nine months. In the case of this patient, there is no clear evidence of hypomanic episodes; however, there is a report of unusual moods during his time at university. Since there are no relatives available to provide further details, the suspected diagnosis is dysthymia and double depression or type II bipolar disorder.

## 3. Discussion

Aripiprazole is a drug approved for use in mania and as a maintenance therapy for the management of bipolar mood disorder. Studies have shown the drug to be useful for the treatment of depression when combined with antidepressants; however, cases have been described in the literature in which significant akathisia developed. In the two cases described here, aripiprazole was associated with lamotrigine and other antidepressants. In the first case, the patient was taking a high dose of lamotrigine (400 mg), while the second patient was using a low dose (100 mg). The prevalence of akathisia in patients with bipolar mood disorder in use of aripiprazole is high, and association of aripiprazole with lamotrigine appears to increase the prevalence of akathisia [8, 9]. In the clinical cases reported here both patients were in use of aripiprazole in combination with lamotrigine and antidepressants, in one case duloxetine and mirtazapine and in the other bupropion. The incidence of akathisia reported in patients with treatment-resistant depression using antidepressants and aripiprazole, even at a low dose, was high [15]. The combination of aripiprazole, the antipsychotic most associated with akathisia, with lamotrigine and antidepressants may have increased the risk of akathisia, thus resulting in the clinical conditions observed in these two cases.

In addition to akathisia, the patients reported a significant worsening of anxiety and the onset of suicidal thoughts, with one attempting defenestration when the dose of aripiprazole was increased to 15 mg. There have been three case reports of suicidal ideation and suicide attempts after patients began using aripiprazole [16–18]. There have also been reports of worsening positive symptoms and disinhibition [19]. With respect to anxiety, we were unable to find any reference to aripiprazole worsening or provoking symptoms of anxiety. The sensation of restlessness and lack of motor control generated by akathisia may alone account for the increased subjective feeling of anxiety. However, in the cases described in this report, anxiety may be associated with deterioration in the patient's general clinical status or may even be one more side effect of the pharmacological association.

Although double-blind controlled studies have been conducted on the use of aripiprazole for the treatment of patients with mood disorder, it may be prudent to exercise caution when prescribing this drug for use in that particular population. Monitoring for the onset of akathisia, increased anxiety, and suicidal thoughts is relevant, particularly in those patients taking multiple drugs.

## 4. Conclusion

In daily practice, physicians must decide how to best manage the treatment of individual cases, which may differ from those encountered in clinical trial settings. When using aripiprazole to treat a patient with mood disorder, adding lamotrigine could be risky. If unavoidable, patients should be closely monitored for the possible development of akathisia, agitation, unbearable anguish and, most importantly, suicidality.
